# Free establishment of primary health care providers: effects on geographical equity

**DOI:** 10.1186/s12913-016-1259-z

**Published:** 2016-01-23

**Authors:** David Isaksson, Paula Blomqvist, Ulrika Winblad

**Affiliations:** 1 Department of Public Health and Caring Services, Health Services Research, Uppsala University, Box 564, 751 22 Uppsala, Sweden; 2 Department of Government, Uppsala University, Box 514, 751 20 Uppsala, Sweden

**Keywords:** Patient choice, Equity, Marketization, Sweden, Primary health care, Geographic location

## Abstract

**Background:**

A reform in 2010 in Swedish primary care made it possible for private primary care providers to establish themselves freely in the country. In the former, publicly planned system, location was strictly regulated by local authorities. The goal of the new reform was to increase access and quality of health care. Critical arguments were raised that the reform could have detrimental effects on equity if the new primary health care providers chose to establish foremost in socioeconomically prosperous areas.

The aim of this study is to examine how the primary care choice reform has affected geographical equity by analysing patterns of establishment on the part of new private providers.

**Methods:**

The basis of the design was to analyse socio-economic data on individuals who reside in the same electoral areas in which the 1411 primary health care centres in Sweden are established. Since the primary health care centres are located within 21 different county councils with different reimbursement schemes, we controlled for possible cluster effects utilizing generalized estimating equations modelling. The empirical material used in the analysis is a cross-sectional data set containing socio-economic data of the geographical areas in which all primary health care centres are established.

**Results:**

When controlling for the effects of the county council regulation, primary health care centres established after the primary care choice reform were found to be located in areas with significantly fewer older adults living alone as well as fewer single parents – groups which generally have lower socio-economic status and high health care needs. However, no significant effects were observed for other socio-economic variables such as mean income, percentage of immigrants, education, unemployment, and children <5 years.

**Conclusions:**

The primary care choice reform seems to have had some negative effects on geographical equity, even though these seem relatively minor.

**Electronic supplementary material:**

The online version of this article (doi:10.1186/s12913-016-1259-z) contains supplementary material, which is available to authorized users.

## Background

Equity in access to health care services is a central policy goal in most publicly funded health care systems. Access is a complex concept that can be understood as a fit between the demand for health care services among the population and the supply of care by providers. To be accessible, health care services need to be affordable, contain the appropriate forms of treatment in relation to medical needs and be physically situated so that patients can get to them [1, 2]. An important component of access is *geographical accessibility*, which can be understood as the distance, or travel time, between patients and health care providers. Equity in geographical accessibility in its simplest form can be understood as all citizens having a similar, or minimum, travel distance to the nearest health care provider [3].

Inequity in geographic access to health care is a well-known problem in many developing countries, where health care services are often underdeveloped, especially in rural areas, and physical infrastructure such as roads and means of transportation may be lacking [4, 5]. Less obvious, inequity in geographical access to health care is also prevalent in many rich countries, despite the existence of well-funded, universalistic health systems [6–9]. The increased recognition of this policy challenge in developed countries is underpinned by studies demonstrating the relationship between the geographical proximity of care, utilization of care services and health outcomes [10–13].

Most of the previous research on inequity in geographical access to health care is focused on differences between regions, identifying, in particular, rural areas as sites of poor geographical accessibility to both primary and secondary health care services [14, 15]. The challenge of providing access to health services to populations in rural, or remote, areas has been extensively discussed in the literature [16, 17]. Generally, it is recognized that this requires special forms of government intervention such as planning the location of care providers, providing extra resources, encouraging cooperation rather than competition between providers and using innovative technology such as telemedicine [16, 18–20].

In addition to studying regions, previous research on geographical access to health services has also looked at the spatial location of health care facilities in neighbourhoods, or areas that are more limited with clusters of residents [21]. This strand of research typically focuses on socio-economic population characteristics such as income, education, and employment status in different neighbourhoods, comparing proximity to health care facilities on the part of residents within them. A wide range of studies demonstrates the impact of neighbourhood residence on health-related lifestyle, utilization of health care services and health outcomes, for instance with regard to infant mortality, self-rated health and heart disease [22–26]. It has been demonstrated, moreover, that further distance to a health care provider in some neighbourhoods resulted in reduced utilization of care services and area-based inequities in health status [11, 27, 28]. This point to neighbourhood residence being a factor in and of itself that contributes to inequities in health, adding to the general socio-economic factors that typically lead to poorer health status among disadvantaged groups. Hence, neighbourhoods and the way they contribute to inequity in geographical access to health care services are important for understanding the creation of inequities in health status within a country or region. Inequity in geographical access to health services has been demonstrated both in relation to secondary and primary care services, even though proximity to primary care services tends to be more evenly distributed between neighbourhoods than to secondary health care services [11]. Even so, proximity to primary care services may be regarded as being of special importance in reducing inequities in health outcomes, as primary care providers often serve as gatekeepers to secondary and preventive services and represent a level of care often used more by lower-income groups than those with higher incomes [29, 30]. Furthermore, reduced access to health care services due to geographic remoteness has been shown to have a stronger negative effect on utilization of such services among groups with lower socio-economic status and poor medical health [31, 32]. Hence, while proximity to a primary care provider may be especially important for the less advantaged, the neighbourhood where they live may have the detrimental effect of offering longer distances to such providers than for other social groups.

Why, then, does the geographical distribution of health care facilities tend to vary, not only between regions but between neighbourhoods in the same city? For primary care services, the answer to this question has typically been sought in studies that examine localization decisions on the part of individual primary care physicians, or GPs. Economists have debated whether physicians should be seen foremost as profit-maximizing, seeking the most densely populated areas where there can be expected to be more customers, or the socially more privileged areas, where health care needs can be expected to be lower and treatments less costly. Other theories suggests that localization decisions on the part of physicians does not only reflect expected income, but preferences in terms of lifestyle, leading to a general desire for urban areas with a wider availability of leisure and cultural activities as well as better schools [7, 33, 34]. In this sense, physicians can be seen as utility-maximizers rather than simply profit-maximizers [35]. Also the age and social background of physicians have been known to play a role, as younger physicians and physicians who grew up in rural areas have been shown to be more willing to move to rural areas when they start a practice [35]. It can be noticed, however, that some of the economic drivers behind localization decisions on the part of physicians, such as expected income due to population density and population health, may not apply in cases where primary care physicians are salaried employees, which has become more common in, for example, the UK in recent years [7].

In addition to studies examining the localization decisions of physicians, there are also studies which investigate the effects of regulatory measures by governments who try to counter-act the uneven distribution of primary care physicians across regions and neighbourhoods. Goddard et al. [7] identifies three main ways in which governments can do this: to increase the supply of physicians in general, hoping that the increased competition will drive some of them to seek out areas with fewer pre-existing practices; to restrict the entry of new physicians into areas which are seen as having an over-supply of physicians already; and finally to use weighted reimbursement formulas or other financial incentives in order to reward physicians who establish themselves in less privileged or more scarcely populated areas. On the basis of the evidence from the UK, where these strategies were practiced in the years 1974–2006, the authors conclude that none of them appear very effective [7]. Studies from other countries point to similar results [1, 6]. The question remains, then, how the goal of equity in geographic access to health services is best promoted.

The case of Sweden is different from most other countries when it comes to the methods used to promote equity in geographical access to primary care. While having an NHS-type, tax-based health care system that in many ways resembles the British system, Sweden, in contrast to the UK and virtually all other countries outside Scandinavia, also has a predominantly public system for providing primary care services. Guided by the ideal of ensuring equitable access to primary care for all residents in the country, irrespective of area of residence, Sweden developed in the post-war era a system of carefully planned allocation of so-called primary health care centres (*PHCCs*) with a multi-disciplinary team of health professionals, operated directly by local public authorities. Hence, primary care physicians have been predominantly salaried employees in Sweden, and the establishment of primary care facilities has been the result of public planning, rather than decisions by self-employed physicians. This situation changed drastically in 2010, however, when a reform was introduced which abolished all restrictions on private entry into the primary care sector. The so-called primary care choice reform (PCCR), with the dual motive of providing better conditions for private entrepreneurship in the primary care sector and offering patients more choice between private and public providers, gave private providers the right to establish and be financially reimbursed on the same conditions as public providers anywhere in the country. Proponents argued that this would increase the total number of primary care providers, hence improving access to primary care generally within the system, while critics feared that the establishment of new private providers would increase inequalities in access to health care as these providers could be expected to target foremost privileged areas [36].

So far, two main evaluations of the Swedish reform have been undertaken which examine its effects in terms of geographic access. The first, carried out by a team of health economists, examined establishment patterns of new privately operated primary health care centres in Stockholm County Council, the largest health region in Sweden. The study showed that the newly established private providers spead themselves quite evenly between different residential areas within the region, seemingly irrespective of differences in average income levels between them [37]. A drawback of the study, however, was that the residential areas used in the analysis were relatively large and included several different types of neighbourhoods. Therefore, it is hard to draw any firm conclusions regarding effects on equity in geographical access between neighbourhoods from the study. The second evaluation, carried out by the Swedish National Audit Office in 2014, examined effects on geographical access to primary health care services of the PCCR by dividing the entire country into small areas, comparable to neighbourhoods. The findings indicate that geographic access to primary care had improved more in socio-economically priviledged areas [38]. Hence, evaluations carried out so far of the Swedish PCCR have found varying results with regard to localization patterns of new private care providers. They are also difficult to compare, as the first study only examines one county council. Thus, it is still unclear what the effects of the PCCR have been in terms of geographic access to primary care in Sweden.

In the present study, we investigate what effects the PCCR has had on geographical equity within the Swedish primary health care system by using data from all county councils. In addition, we use statistical models to control for county council effects, which imply that we can examine establishment patterns of private care providers without the distorting effects of regulations and reimbursement formulas in individual county councils. In this sense, the present study has both a broader scope than the Stockholm study and a stronger methodological design than the study by the National Audit Office.

The rest of the paper is organized in four sections. It proceeds with a description of the Swedish health care system and the 2010 primary care choice reform. Next, we present the methodology of the study, followed by an analysis of the empirical data and presentation of the findings. The final section of the paper discusses the results and situates them in the context of previous research on geographical accessibility.

### The Swedish health care system and the primary care choice reform

The Swedish healthcare system is governed primarily at the regional level by 21 relatively autonomous bodies, the so-called county councils. These are governed by boards which are locally elected every fourth year. The county councils are responsible for the provision and funding of health care services and have the right to make independent decisions on a wide range of policy issues, including the allocation of health services between primary and secondary care, ways to reimburse care providers, public health interventions and the overall organization of the local health care system. The budget is fixed per year and funding comes primarily from local income taxation (around 80 %); in addition to this, 15–20 % of the funding originates from national government grants and, to a smaller extent, patient fees. The county councils are obliged, through the Swedish Health and Medical Service Act, to guarantee that all citizens in the county receive high quality health care services. The county councils can also choose to contract with private care providers.

Even if the organization of health care is decentralised, the internal structure of the health care system looks quite similar in the various county councils. Medical care is usually divided into three levels: primary, hospital, and regional care (including highly specialized services). As noted in the introduction, primary care is mostly provided at primary health care centres. Unlike primary care in most other countries., which is structured around individual GPs [39], the Swedish primary care centres are multidisciplinary and generally consist of 4–10 GPs, nurses, physical therapists and dieticians. In addition to medical examinations, treatment and basic medical care, the centres also offer public health and preventive services as well as health counselling [40]. Many of the primary health care centres also provide prenatal- and maternity care. Patients with more advanced health problems are referred to specialist care at hospitals or private specialist clinics. Even though most county councils do not apply a formal gate-keeping system, a referral from a GP often leads to quicker assessment at hospital or specialist level.

Since the post war-era, when the modern health care system in Sweden developed, county councils have been in charge of planning and setting up new health care centres. Generally, decisions on where to establish public health centres have been based on population statistics on health care needs and the goal of close proximity to a local health centre for all residents, regardless of level of income or residential area. According to a national regulation from 2000, the goal should be *a minimum level of one GP per 1500 patients*. Even if the overwhelming majority of primary care physicians have been employed at public health care centres, private providers were formally allowed in the system. The locations of these, however, had to be authorized by the county councils. Hence, in the Swedish health care system, there has been firm regulatory control over the supply of primary care providers and their establishment has been publicly planned solely on the basis of estimated population needs.

In January 2010, the governance of the primary care system in Sweden changed significantly as a centre-right government launched a reform known as the Primary care choice reform (PCCR). The reform consisted of two parts: (1) that citizens get to choose at what health care centre they want to be listed, and (2) that all health care providers who meet certain quality requirements, set up by the counties, may establish themselves anywhere in the county [41]. A central motive behind the reform was to create a more market-based system, where competition between private and public care providers in combination with free choice of where to enlist on the part of patients should provide better conditions for private entrepreneurship and increase the access to services on the part of patients [36]. The reform resulted in a rapid expansion of the share of private, predominantly for-profit, providers within the system testifying to the strong financial incentives for new establishments [42].

The PCCR implied a substantial change in the previous primary care model that had relied on a bureaucratic steering where the county councils managed and organised health care services directly and planned their distribution on the basis of assessed needs [43, 44]. With the introduction of the reform, new private providers can freely decide where to locate and have the right to receive payments on the same terms as public health centres; usually based on a mix of capitation and fee-for service. This implies that the county councils lose control over how resources are distributed within their respective areas, and that the connection between assessed health needs and the location of primary care providers is lost. However, it should be noted that the county councils still retain the right to locate public primary health care centres and to regulate the new health care markets by specifying the range of health care services that should be offered by the primary care providers. This has led to the fact that in most county councils the main form of primary care provider is still a relatively large multi-disciplinary health centre offering a wide range of health-related services, including maternity care, child care and psychological counselling. Likewise, the vast majority of physicians within the system are still salaried employees, even in the privately owned centres. Most of these are owned by large commercially-oriented chains [38].

#### Reimbursement of the primary health care centres

Due to the decentralized Swedish health care system, each county council has its own reimbursement model for health care. Hospital care is financed chiefly through global budgets with some elements of DRG-based per-case payment. However, the reimbursement models for primary care have changed from a budget model to a capitation model due to the 2010 reform. Basically, the primary health care centres receive a fixed sum per listed patient, a so-called capitation fee and an additional payment per patient visit. At present, the capitation varies from 40 to 100 % of the total reimbursement in the county councils, the rest being predominantly payments per visit. Even if there is substantial variation, the majority of county councils use a reimbursement system which is heavily shifted towards capitation payments [43]. The most conspicuous change following the PCCR, however, is that money now “follows the patients”, meaning that if a PHCC does not attract any patients they will not receive any funding. Thus, funding is not guaranteed, but dependent on how well the providers manage to attract new patients [36, 40]. Hence, in the current system, public and private health care centres compete on the same terms and public PHCCs have no specific advantage.

Common to all counties is also that they use some form of risk-adjusted payment built into the capitation fee in order to avoid patient selection. One of the more commonly used methods involves a risk adjustment based on age, health status and socio-economic characteristics such as employment, income and education level of the listed patients. Even if all county councils use risk adjustment, the degree to which it is employed and specific factors weighted varies. For instance, in 2013, 18 counties out of 21 compensated for age, giving a higher capitation for elderly patients. However, advanced age is not synonymous with illness, which is why caregivers may still try to attract older, healthy individuals. To avoid this problem, several counties have chosen to adjust compensation based on diagnoses by introducing so-called Adjusted Clinical Groups (ACG) index. In 2013, ten counties out of 21 also based their reimbursement on the listed patients’ health status and 16 compensated for socio-economic characteristics. Also, in 14 of the county councils the providers received extra payments if they located in remote areas [45].

The aim of this study is to examine what effect the primary care choice reform has had on the geographical distribution of primary care providers. Where have new, predominantly private providers, established themselves and to what extent have their localisation decisions added to inequities between neighbourhoods in geographical access to care services? To investigate this, we utilize a cross-sectional data set containing socio-economic data of the geographical areas of all primary health care centres in Sweden.

## Methods

The empirical analysis in the study is carried out by analysing the character of the new providers and where they have established themselves in the 21 county councils. The basis for the design of the study is to analyze socio-economic data from 2013 on individuals who reside in the same electoral areas in which the 1411 primary health care centres in Sweden are located. In this sense the electoral areas can be seen as corresponding to the concept of neighbourhood [27]. The socio-economic data on the persons living in these areas is gathered from Statistics Sweden (*SCB*). The data consists of the following variables:

- Mean income
- Percentage of people born outside Western Europe and North America
- Percentage of single parents living with children under 18 years of age
- Percentage of people who are low-educated (<10 years schooling)
- Percentage of people who are unonemployed or enrolled in public programs
- Percentage of people over 65 years of age living alone
- Percentage of people younger than 5 years of age

The variables are similar to variables used in previous studies of the effects of patient choice reforms and are meant to capture socio-economic status that is related to expected health care needs - e.g., people that are less educated generally have lower health status [46, 47] and people who are unemployed are to a higher degree likely to suffer from depression and anxiety [48].

One of the previous studies used the percentage of people over 65 years to capture socio-economic status [38]. We argue that the percentage of people over 65 years living alone is a more appropriate variable since older adults living alone have poorer health than older adults living with a partner [49] and thus are likely to have higher health care needs.

Socio-economic data is gathered on all people living in the same electoral area as each primary health care centre. The number of citizens in each electoral area varies from 100 to 10,000, making these units of analysis equivalent to what in the literature is referred to as neighbourhoods [27]. We also use descriptive data from all primary health care centres, regarding when they started up, ownership structure and if they are affiliated with other primary health care centres.

Since all primary health care centres belong to a specific county council and thereby are affected by different reimbursement systems as well as different county characteristics, we will group the primary health care centres in a secondary level variable according to which county council they belong to.

To examine whether there are any differences regarding where old and new primary health care centres choose to establish themselves after the PCCR, we use comparisons of means and t-tests. In our main models, when controlling for possible cluster effects of county councils and municipalities, generalized estimating equation (GEE) models are used. The GEE-models are an extension of generalized linear models constructed to produce more solid results with correlated, or clustered, data [50, 51]. This method is used since the socio-economic variables can be correlated within certain municipalities and/or county councils and a regular OLS regression would thus risk violating the independence assumption [50, 52]. One of the main reasons for choosing GEE rather than HLM is that GEE in general can be considered to be more robust against model misspecifications than other types of multilevel models such as HLM [53]. In the GEE models, we use municipalities as clusters. One could argue that county councils would be a better choice for clustering since it is the county councils who design the primary care systems. However, when fitting the same models with county councils, we get similar results but with a slightly worse model fit. To select the best regression model the corrected quasi-likelihood under the independence model criterion (QICC) was used [51].

All statistical analyses were performed using R, v. 3.2.0 [54]. When performing the GEE-models the package ‘geepack’, v. 1.2-0 [55] was used.

## Results

Our first question concerns what types of primary health care centres (public or private) are being established and closed down after the primary care choice reform. Privately owned primary health care centres (PHCCs) can be expected to have higher demands for profitability than publicly owned PHCCs [56]. Thus, if a large majority of the new primary health care centres are private, there is probably a higher risk of patient selection.

Table 1 shows whether each primary health care centre – private or public – was established before or after the PCCR. Since each county council implemented the reform at different times, the dates for the reform start vary from 2007, when the primary care choice reform was introduced in one of the counties (Halland), to 2010 when it became mandatory for all county council to adopt a primary care choice system.

**Table 1:** Ownership status of PHCCs established before and after the introduction of the primary care choice reform

	Public	Private	Total
Old PHCC	762 (70 %)	327 (30 %)	1089 (100 %)
New PHCC	15 (5 %)	270 (95 %)	285 (100 %)
Closed	−32 (48 %)	−34 (52 %)	−66 (100 %)
Total	745 (57 %)	563 (43 %)	1308 (100 %)

As shown in Table 1, 285 new PHCCs have started up since the reform was implemented. Private companies owned most of them, 95 %, while merely 15, or 5 %, of the new PHCCs were publicly owned and run by the county councils. In close proximity to the reform, a relatively large number of PHCCs closed down; some of those were private PHCCs that chose to shut down their business shortly before the primary care choice reform was introduced. This could possibly be explained by increased demands on the scope of medical services that were required in some of the counties, e.g. maternity care and child care [41]. Apart from that, both private and publicly owned PHCCs have closed down after the introduction of PCCR, for example due to too few enlisted patients. Finally, our data show that a great majority of the new providers are privately run for-profit PHCCs.

The primary care choice reform, however, has had varying effects in different county councils. Table 2 below shows the development in all county councils.

**Table 2:** Old and new PHCCs in all county councils

County council	Population (millions)	Old PHCC	New PHCC	Total PHCC	% New
Blekinge	0.2	23	3	26	12 %
Dalarna	0.3	31	5	36	14 %
Gotland	0.1	9	0	9	0 %
Gävleborg	0.3	39	7	46	15 %
Halland	0.3	38	16	54	30 %
Jämtland	0.1	27	2	29	7 %
Jönköping	0.3	46	18	64	28 %
Kalmar	0.2	44	1	45	2 %
Kronoberg	0.2	29	7	36	19 %
Norrbotten	0.2	35	5	40	13 %
Skåne	1.3	141	34	175	19 %
Stockholm	2.1	179	60	239	25 %
Södermanland	0.3	23	7	30	23 %
Uppsala	0.3	46	12	58	21 %
Värmland	0.3	33	8	41	20 %
Västerbotten	0.3	36	5	41	12 %
Västernorrland	0.2	39	7	46	15 %
Västmanland	0.3	32	7	39	18 %
Västra Götaland	1.6	164	76	240	32 %
Örebro	0.3	32	1	33	3 %
Östergötland	0.4	43	4	47	9 %
Total	9.6	1089	285	1374	21 %

As shown above, there is a large variation between county councils when it comes to the effects of the establishment of new private providers. One county council (Gotland) has no new establishments of primary health care centres whereas Västra Götaland and Halland have seen an increase in the numbers of PHCCs of over 30 %. In absolute terms, Västra Götaland and Stockholm are the county councils with the largest increase in PHCCs, with 76 and 60 new primary health care centres respectively. These are both large city regions. Additional file 1: Appendix also shows that there is substantial variation between county councils when it comes to structural variables such as number of inhabitants, mean income, immigrants, and percentage of population over 65 years.

To summarize, the primary care choice reform has resulted in over 270 new private primary care providers opening up new practices in different parts of the country – virtually all of which are owned by private, for-profit companies. This is an increase of 45 % in the share of private primary care providers in the system. A majority of these are for-profit providers leading to a different type of market structure than in the current system. There has also been a clear tendency for new private care providers to establish themselves foremost in urban areas, like the large city regions of Stockholm, Gothenburg, and Malmö.

Our second question sets out to investigate if new primary health care centres establish themselves in socio-economically more prosperous areas. In Table 3 we have compared different socio-economic values between old PHCCs and new PHCCs established after the primary care choice reform. The analysis shows mean socio-economic values based on all people living in the same electoral area where each PHCC is located. Data are presented on each socio-economic variable for old and new PHCCs.

**Table 3:** Comparisons of means of socio-economic variables based on whether the PHCC was established before or after the primary care choice reform. Significance values shown for a 2-sided *t*-test for equality of means

	Old PHCCs	New PHCCs	Sig. (2-tailed)
% Immigrants from outside of Western Europe	7.1 %	7.8 %	.142
% Single parents	3.0 %	2.8 %	.000
% >65 years and living alone	11.3 %	9.9 %	.000
Mean income/year (kSEK)	201.1	211.0	.001
% Unemployed or in public programs	11.4 %	10.6 %	.001
% Younger than 5 years	5.4 %	5.5 %	.126
% Less-educated 25–65 years	7.2 %	6.5 %	.001

As shown in Table 3, primary health care centres that established themselves after the PCCR tend to do so in areas which have higher socio-economic status than what was the case for primary health care centres that already existed before the reform. This is the case for the variables *Unemployed or in public programs*, *Low-educated 25 – 65 years*, *Single Parents, > 65 years and living alone* and *Mean income.* For example, the mean percentage of unemployed in areas where old primary health care centres are established is 11.4 % while the corresponding number for new PHCCs is 10.6 %. The mean income in areas where old PHCCs are established is 201 kSEK while the corresponding number for new PHCCs is 211 kSEK, meaning that new PHCCs are located in areas with higher incomes. One exception exists, however – the new PHCCs are to a higher degree located in areas with a higher percentage of *Immigrants from outside of Western Europe*. The difference is small and is not statistically significant with a significance level of 95 %.

A possible bias of the results in the *t*-test can be underlying differences between the county councils, both in regard to socio-economic variables or the way county councils risk adjust the compensation according to socio-economic differences, but also to the degree of established PHCCs after the reform. This type of cluster effect is also likely to be found at the municipality level. To control for the cluster effect, we will fit GEE models with municipalities used as the cluster.

The results from the GEE models are found in Table 4.

**Table 4:** GEE-models with a binary dependent variable where 0 = old PHCC, 1 = new PHCC

	Model 1	Model 2	Model 3	Model 4	Model 5	Model 6
Intercept	−1.64^***^	−1.62^***^	−1.58^***^	−1.53^***^	−1.50^***^	−1.49^***^
% Immigrants outside Western Europe	−0.059				0.179^*^	0.151
% Single parents		−0.323^***^			−0.248^**^	−0.282^***^
% >65 years and living alone				−0.250^***^	−0.280^**^	−0.217^**^
Mean income			0.118		−0.005	
% Unemployed or in public programs					−0.068	
% Younger than 5 years					−0.124	
% Less-educated 25–65 years					−0.116	−0.150
QICC	1440	1413	1423	1400	1382	1377

Sign.codes: ‘***’ 0.001, ‘**’ 0.01, ‘*’ 0.05, ‘.’ 0.1 ‘. Correlation: Structure = exchangeable. Number of clusters = 290 (all municipalities). All included variables are standardized

Table 4 shows the results from our different GEE-models using municipalities as clusters. In the first model we only looked at *Percentage of immigrants* as an estimator. After controlling for the cluster effect using the GEE-model we see no significant difference in where old and new PHCCs are located. Model 2 and 4 show that both *Single parents* and *Older adults* living alone are more uncommon in areas where new PHCCs are located when controlling for municipality effects. Interestingly we find no significant difference in the mean income between areas where PHCCs established after the primary care choice reform and before.

In model 5 we included all socio-economic variables that theoretically could be likely to increase the need for health care services. The same significant effects are seen as in model 2 and 4, but there is also a significant effect of the percentage of immigrants. In other words, when controlling for clusters and all other variables, PHCCs established after the PCCR are to a greater extent located in areas with a higher percentage of immigrants.

In model 6 we have included the estimators that gave the best model fit according to the QICC-value. The results show that both percentages of single parents and older adults living alone are still significant but that the significance of the percentage of immigrants from outside of Western Europe has disappeared even though the sign is still positive.

To summarize, the results show that the primary care choice reform seems to have some negative effects on geographical equity. When controlling for the effects of clusters, PHCCs established after the primary care choice reform are located in areas with statistically significantly fewer older adults living alone as well as fewer single parents. However, there are no significant effects for the other studied variables, such as education, immigrants, young children or mean income.

## Discussion

In recent decades, equity in geographical access to health care services has been a growing concern among policy makers in both developed and developing countries. At same time, many countries have introduced reforms aimed at marketizing health care provision. Driven by a desire to reduce costs and allocate resources in a more efficient manner, political leaders have sought to increase competition and open the door to more entrepreneurial care providers, such as for-profit companies [41, 57]. Privatization of health care provision can have positive effects in terms of efficiency and rationalization but may also lead to undesirable consequences, such as provider selection of patients and a further increase in the inequitable access to health care services [36, 58, 59]. One way in which equity may be undermined is through patient selection through geographical establishment. If a provider establishes in a socio-economically more prosperous area, the patient population is likely to be healthier and consume simpler types of health care services than in areas with lower socio-economic status. In a publicly regulated health care system, where open patient selection through rejection of certain patient groups is prohibited, establishment of care facilities may in fact be one of the most important mechanisms for patient selection. Despite this, it has only rarely been systematically examined in the literature.

In this paper we have investigated whether there are any visible patterns of patient selection through uneven geographical establishment after the introduction of the primary care choice reform, which allowed free entry for private providers. The results of the study indicate, first, that almost all primary health care centres that were established after PCCR are privately owned and for-profit. Secondly, our data show that the establishments of new private providers are unevenly distributed between the county councils, with most of the new providers choosing to locate in the largest cities.

Third, our findings show that there is also a pattern of uneven geographical establishment within the county councils, or between neighbourhoods. In the first step of the analysis, we looked at the effect without controlling for county council effects. We could see that the areas, or neighbourhoods, where new privately operated primary health care centres were located had lower percentages of less educated and unemployed persons, and a lower percentage of older adults living alone. Income levels were also generally higher. However, when controlling for the cluster effects of the county councils by using GEE-models, most of the studied variables were no longer statistically significant. The only significant statistical effect left was for the percentage of people over 65 years living alone, and percentage of single parents. As these groups are known to have high health care needs, this points to some, albeit limited, patient selection going on after the reform.

In this sense, our results confirm the findings of the study by the Swedish Audit Society that there were some negative effects on geographical equity after the introduction of the 2010 primary care choice reform. The effects do not appear to be very large, and they concern only some indicators of socio-economically less advantaged areas. How are these relatively small effects on geographical equity to be interpreted? In a Swedish context, with a strong political emphasis on equal distribution of health care resources and a long history of planned health care services, the results can still be seen as noteworthy, as even a small reduction in geographical equity in access to care services represents a threat to the ideal of a universalistic and egalitarian health care system. In an international context, where private care providers are considerably more common and economic drivers can be expected to play a larger role, the results may be less surprising.

Furthermore, the question can be raised as to why the uneven establishment pattern is not more pronounced, given the profit motive on the part of most of the new private providers. Why have private providers in Sweden not established themselves in prosperous areas to a larger extent? Even though we have seen a marketization of the Swedish health care system in recent years, there is reason to believe that the county councils are still able to exercise some influence over the strategies and choices of care providers with regard to where they establish and what kinds of patients they seek to attract. The main tool in this regard is the weighted capitation system, which serves to counter-act incentives to select the healthiest patient groups.

As noted in the introduction, the literature has identified three different types of strategies to be used by government authorities’ when they seek to promote a more even distribution of primary care providers across regions and neighbourhoods [7]. The first was to restrict entry into areas with an already high number of care providers. This was the strategy Swedish county councils used prior to the introduction of the PCCR. However, this strategy is no longer possible given that the PCCR ensures the free entry of providers. The second strategy was to increase the number of primary care physicians within the system in the hope that they will spread more evenly across regions and neighbourhoods. This strategy can be seen employed through the PCCR, but has apparently not generated the desired result. The third strategy, to risk adjust the reimbursement formulas in order to make it more attractive to locate in disadvantaged and remote areas, seems to have been used to a large extent in the Swedish case, and is probably an important explanation as to why private care providers have not exercised more patient selection through their location decisions. In this sense, this strategy can be seen as successful in the Swedish case. Unfortunately, the design of our study does not help us to understand which combination of risk adjustments in reimbursements leads to the most equal geographical distribution of health care providers. Thus, one of the main policy implications of the study is the need to evaluate how a particular reimbursement design affects the behaviour of profit-driven health care providers. If, as can be seen in this study, there is a tendency of such providers to establish predominantly in areas with lower health care needs, further adjustments in the reimbursement formulas to compensate even more effectively for health risks might be a way forward if the goal of geographical equity is to be promoted.

## Conclusion

The results show that the primary care choice reform has had some negative effects on geographical equity. When controlling for the effects of clusters, primary health care centres established after the new reform are located in areas with significantly fewer older adults living alone as well as fewer single parents. However, none of the other variables, such as education, income, employment, or children in the household, shows any significant effects, leading to the conclusion that the county councils, to some extent, have managed to buffer against unequal distribution of private providers.

## Additional file

Additional file 1: Appendix. (DOCX 21 kb)
