# Supplementary material for: Free establishment of primary health care providers: effects on geographical equity
Source: BMC Health Serv Res. 2016 Jan 23;16:28. doi: 10.1186/s12913-016-1259-z (PMC4724405; doi:10.1186/s12913-016-1259-z)
Supplement: Additional file 1: Appendix. — (DOCX 21 kb) [file 12913_2016_1259_MOESM1_ESM.docx]

| County | Population | % of pop. 65+ | % with low income | % unemployed | Mean income  (SEK) | % on welfare | % of pop. born outside Scandinavia and EU | % low-educated  (20–40 y.o.) |
| --- | --- | --- | --- | --- | --- | --- | --- | --- |
| Blekinge | 154,157 | 23.4 | 21.4 | 13.6 | 234,360 | 4.3 | 5.4 | 9.2 |
| Dalarna | 278,903 | 23.6 | 20.6 | 11.8 | 240,912 | 5.3 | 5.4 | 10.8 |
| Gotland | 57,255 | 23.9 | 21.7 | 11.7 | 224,529 | 4.3 | 2.3 | 11.1 |
| Gävleborg | 279,991 | 23.2 | 21.0 | 15.0 | 237,439 | 5.9 | 7.1 | 11.2 |
| Halland | 310,665 | 21.2 | 16.5 | 10.3 | 255,641 | 2.7 | 4.1 | 8.0 |
| Jämtland | 126,765 | 22.8 | 21.0 | 12.9 | 233,546 | 4.1 | 4.3 | 8.5 |
| Jönköping | 344,262 | 20.6 | 19.1 | 10.6 | 250,577 | 4.9 | 6.8 | 8.5 |
| Kalmar | 235,598 | 24.2 | 19.7 | 11.2 | 235,309 | 3.5 | 4.7 | 9.8 |
| Kronoberg | 189,128 | 21.1 | 20.5 | 12.0 | 241,585 | 5.0 | 7.4 | 8.1 |
| Norrbotten | 249,987 | 22.8 | 16.5 | 12.2 | 252,174 | 3.1 | 3.9 | 9.8 |
| Skåne | 1,288,908 | 19.4 | 24.8 | 12.8 | 231,046 | 5.3 | 8.0 | 8.9 |
| Stockholm | 2,198,044 | 15.7 | 18.6 | 10.1 | 276,611 | 2.8 | 12.4 | 8.8 |
| Södermanland | 280,666 | 22.1 | 21.5 | 14.1 | 241,610 | 7.1 | 9.4 | 11.9 |
| Uppsala | 348,942 | 18.3 | 19.7 | 9.0 | 252,913 | 3.8 | 8.0 | 8.1 |
| Värmland | 274,691 | 23.2 | 23.6 | 12.8 | 231,018 | 4.6 | 4.6 | 9.2 |
| Västerbotten | 262,362 | 20.4 | 20.1 | 11.4 | 244,764 | 4.8 | 5.3 | 8.7 |
| Västernorrland | 243,061 | 23.4 | 19.4 | 11.4 | 246,427 | 3.5 | 5.4 | 7.5 |
| Västmanland | 261,703 | 21.6 | 19.0 | 14.0 | 244,632 | 4.5 | 9.3 | 10.3 |
| Västra Götaland | 1,632,012 | 19.1 | 20.3 | 13.3 | 248,729 | 5.3 | 8.0 | 11.5 |
| Örebro | 288,150 | 21.0 | 21.0 | 13.1 | 240,876 | 5.0 | 7.5 | 9.7 |
| Östergötland | 442,105 | 20.1 | 21.1 | 12.0 | 239,172 | 5.8 | 7.6 | 9.0 |
| Total | 9,747,355 | 19.6 | 20.3 | 11.6 | 248,690 | 4.4 | 8.2 | 9.1 |

Sources: Statistics Sweden, Swedish Association of Local Authorities and Regions, Swedish National Board of Health and Welfare. All data is from 2014.
